# An exploratory study of gut microbiota and serum metabolite profiles in type 2 diabetes patients with sleep disorders

**DOI:** 10.3389/fmicb.2026.1911955

**Published:** 2026-10-12

**Authors:** Jinkun Ma, Xinbin Zhang, Yirao Pan, Xueli Bao, Hua Yi, Jing Hua, Qianqian Mu, Lantian Liu, Chunyu Hu, Dandan Zhao, Sihua Gao

**Affiliations:** 1 Graduate School, China Academy of Chinese Medical Sciences, Beijing, China; 2 School of Traditional Chinese Medicine, Beijing University of Chinese Medicine, Beijing, China; 3 Guang'anmen Hospital, China Academy of Chinese Medical Sciences, Beijing, China; 4 Dongzhimen Hospital of Beijing University of Chinese Medicine, Beijing, China; 5 Department of Endocrinology, Yancheng Third People's Hospital, Yancheng, China; 6 General Outpatient Department, Beijing University of Chinese Medicine Third Affiliated Hospital, Beijing, China

**Keywords:** 16S rRNA gene sequencing, gut microbiota, metabolomics, sleep disorders, type 2 diabetes

## Abstract

**Objective:**

To characterize gut microbiota composition and serum metabolites in T2DM patients with and without sleep disorders.

**Methods:**

This single center cross sectional study enrolled 40 T2DM patients with sleep disorders (Group I), 40 T2DM patients without sleep disorders (Group N) and 30 age and sex matched healthy controls (Group ZC). Fecal and serum samples were collected for 16S rRNA gene sequencing and untargeted liquid chromatography–mass spectrometry metabolomics, respectively. Gut microbiota composition, serum metabolites and metabolic pathways, and microbe–metabolite correlations were analyzed.

**Results:**

Compared with T2DM patients without sleep disorders, T2DM patients with sleep disorders showed reduced gut microbial diversity and evenness, as indicated by lower Shannon, Simpson, and Pielou's evenness indices. Beta diversity analysis showed a small but statistically significant difference in microbial structure between Groups I and N. At the genus level, nominal differences were observed between the two groups, including higher relative abundances of *TM7x, Achromobacter*, and *Pseudomonas* and lower relative abundances of *Eubacterium_eligens_group, Lachnospiraceae_UCG-001*, and *Negativibacillus* in Group I. Metabolomic results revealed 17 nominally differential metabolites between Groups I and N, involving caffeine metabolism and porphyrin and chlorophyll metabolism. Spearman analysis revealed that stercobilin abundance positively correlated with *Lachnospiraceae_UCG-001* (*R* = 0.381, *P* < 0.001, *Q* = 0.095), *Lachnospiraceae_ND3007* (*R* = 0.354, *P* = 0.002, *Q* = 0.121), and *Odoribacter* (*R* = 0.280, *P* = 0.01, *Q* = 0.301).

**Conclusions:**

T2DM patients with sleep disorders showed modest differences in gut microbial community structure and exploratory changes in serum metabolite profiles. Stercobilin and caffeine-related metabolic features may represent exploratory characteristics associated with sleep disorders in T2DM. These findings suggest potential microbiota-metabolite associations and provide a basis for future longitudinal and mechanistic studies.

## 1 Introduction

Type 2 diabetes mellitus (T2DM) is a metabolic disorder characterized by long-term elevated blood glucose levels. The clinical features of T2DM include polydipsia, polyuria, polyphagia, and weight loss (American Diabetes Association Professional Practice Committee, 2025). Additionally, T2DM patients may experience acute and chronic severe metabolic disturbances, infections, microvascular complications, and various other complications. Over the past 40 years, T2DM has become one of the most severe public health issues globally, with the prevalence in China significantly increasing to 10.4% (Society CD, 2021; Zia et al., 2024).

Sleep disorders include insomnia, hypersomnia, sleep apnea syndrome, and restless legs syndrome (Arlington VA, 2013). Increasing evidence indicates a correlation between T2DM and sleep disorders. Due to metabolic disturbances associated with diabetes, the prevalence of sleep disorders in diabetic patients is significantly higher than in the healthy population. Studies have shown that up to 52% of diabetic patients suffer from concurrent sleep disorders (Khalil et al., 2020). Conversely, individuals with sleep disorders are at a 40% increased risk of developing diabetes (Thunyarat et al., 2016). Cohort studies suggest that short sleep duration is a risk factor for T2DM (Antza et al., 2021). However, the pathological mechanisms of sleep disorders in T2DM are poorly understood. This study hypothesizes that there is a bidirectional pathophysiological interaction between T2DM and sleep disorders, in which the gut microbiota and its metabolites may play a key role in this interaction.

The gut microbiota refers to the community of microorganisms that inhabit within the host's intestines, existing in a symbiotic relationship with the host. These microorganisms play a pivotal role in maintaining human health and facilitating normal physiological functions (Liu et al., 2023). The gut microbiota, *via* its metabolic products such as short-chain fatty acids (SCFAs), lipopolysaccharides (LPS), amino acids (AAs), and bile acids, plays a fundamental role in regulating body metabolism and immune homeostasis (Li et al., 2023; Liu et al., 2022; Wu et al., 2023). This microbial community has been implicated in the pathogenesis of a wide range of diseases. Recent basic and clinical research has indicated that gut microbiota is closely related to the development of T2DM and sleep regulation. Modulation of gut microbiota is involved in the development, prevention, and treatment of T2DM (Jia et al., 2023; Qin et al., 2012; Sonnenburg and Bäckhed, 2016; Wen et al., 2025; Zhao et al., 2024). Similar disruptions in gut microbiota can also be observed in individuals with sleep disorders (Jiwei et al., 2024; Yong et al., 2023). On the other hand, supplementation with probiotics can lower sleep quality index scores and improve sleep quality (Yifan et al., 2023). However, the role of gut microbiota in individuals with T2DM who also suffer from sleep disorders remains to be explored. Metabolomics involves the quantitative analysis of small molecule metabolites within an organism, allowing the exploration of the relationship between metabolites and pathological changes in diseases (Huimin et al., 2024). By quantifying and amplifying metabolites, metabolomics can effectively identify differential metabolites and metabolic pathways, thus revealing the related mechanisms of T2DM with sleep disorders at the overall metabolic level. Therefore, this study uses 16S rRNA gene sequencing and metabolomics analysis to compare the difference in gut microbiota and serum metabolites between diabetic patients with sleep disorders and those without. The aim is to identify key differences in gut microbiota and metabolites in T2DM patients with sleep disorders, providing a scientific foundation for understanding the mechanisms and developing clinical interventions.

## 2 Materials and methods

### 2.1 Study design

This study adopted a cross-sectional design involving three groups: T2DM patients with sleep disorders (Group I), T2DM patients without sleep disorders (Group N), and healthy controls (Group ZC). The primary outcome was the difference in gut microbial alpha diversity (Shannon index) between Group I and Group N. Sample size was estimated using G^*^Power software (version 3.1) for a two independent samples *t*-test, as the primary comparison was between the two diabetic groups. With α = 0.05 and power = 0.80, 40 participants per group provides 80% power to detect a minimum effect size of Cohen's *d* ≈ 0.63 (medium to large). This effect size is consistent with those reported in previous gut microbiota studies in T2DM populations. A healthy control group (Group ZC, *n* = 30) was included for baseline reference.

### 2.2 Participants and ethics

The study randomly enrolled 80 patients with T2DM from the Third People's Hospital of Yancheng City from January 2023 to June 2023 and 30 healthy volunteers without sleep disorders from the same community during the same period (Group ZC). The T2DM patients were divided into two groups based on the presence or absence of sleep disorders: the Sleep Disorder Group (Group I) and the Non-Sleep Disorder Group (Group N), with 40 participants in each group. The control group was frequency-matched by age and sex; the smaller sample size was due to recruitment constraints. The study protocol was approved by the Ethics Committee of the Third People's Hospital of Yancheng City (Ethics No.: Ethics Review-2023-02). All participants have provided written informed consent.

### 2.3 Diagnostic criteria

T2DM: (1) Typical symptoms include polydipsia, polyuria, polyphagia, and unexplained weight loss. (2) Random blood glucose ≥11.1 mmol/L or fasting plasma glucose (FPG) level ≥7.0 mmol/L or OGTT 2-h blood glucose level ≥11.1 mmol/L or HbA1c ≥6.5% (Society CD, 2021).

Sleep disorders: patients who are dissatisfied with their nighttime sleep, such as having difficulty falling asleep, sleep maintenance disorders, early awakening, decreased sleep quality, and reduced total sleep time, and whose Pittsburgh Sleep Quality Index (PSQI) score is greater than seven (Mollayeva et al., 2016; Sleep Disorder Group, 2026). In this study, sleep disturbance was defined based on PSQI assessment and therefore does not distinguish specific sleep-disorder subtypes.

### 2.4 Inclusion and exclusion criteria

Inclusion criteria:

For the diabetes group:

- Patients aged between 25 and 80 years, gender unrestricted;
- Meeting the diagnostic criteria for T2DM;
- Understanding and agreeing to participate in this study, with signed informed consent.

For the healthy group:

- Individuals aged between 25 and 80 years, gender unrestricted;
- No abnormalities detected in health check-up results;
- Assessed by the PSQI with a total score ≤ 7, to exclude individuals with sleep disorders;
- Understanding and agreeing to participate in this study, with signed informed consent.

Exclusion criteria:

- Patients with type 1 diabetes, gestational diabetes, secondary diabetes, or uncertain types of diabetes;
- Patients with severe primary diseases of other organs such as the heart, liver, or kidneys;
- Patients with acute diabetic complications, such as diabetic ketoacidosis, hyperosmolar hyperglycemic state, or infections;
- Individuals who have suffered from irritable bowel syndrome, severe diarrhea, or other gastrointestinal diseases in the last 3 months, or who have taken antibiotics glucocorticoids, or other medications which could affect the gut microbiota;
- Individuals who have consumed yogurt within the past week;
- Individuals with a history of gastrointestinal or gallbladder surgery;
- Individuals with psychiatric diseases, infectious diseases, or those who are unable to take care of themselves;
- Individuals participating in other research studies.

Elimination criteria: participants who did not collect samples at the required time or who lost contact.

### 2.5 Information and sample collection

For each participant, basic information (age, gender, height, weight, waist circumference, and duration of diabetes) was recorded, along with the use of glucose-lowering medications.

Participants were instructed to fast for at least 10 h prior to sample collection. Three tubes of fresh venous blood, approximately 3 mL each, were collected from each subject. The blood samples were allowed to clot for 30 min and then centrifuged at 3,000 rpm for 10 min at 4 C. Two of the tubes were used for biochemical and glucose analysis, including fasting FPG, total cholesterol (TC), triglycerides (TG), high-density lipoprotein cholesterol (HDL-C), low-density lipoprotein cholesterol (LDL-C), uric acid (UA), and lipoprotein a [Lp(a)]. The blood in the third tube was used for serum metabolomics analysis. All data were processed anonymously, and the samples were destroyed after the experiment.

Participants were provided with a sealed feces collection kit and a bio-ice pack the day before sample collection. They were instructed to collect 3~5 g of morning feces in a standardized manner, record the sampling time, and deliver the sample to the laboratory within 2 h. Feces samples were aliquoted into three 1.5 mL eppendorf tubes, with 0.5 ml per tube, and stored at −80 C for subsequent gut microbiota analysis. Samples were excluded if they were not delivered in a timely manner or if contamination occurred due to conditions such as hemorrhoid rupture, diarrhea, or anal fissure.

### 2.6 Serum metabolomics analysis

#### 2.6.1 Serum sample extraction

A 100 μL blood sample was placed into an EP tube, and 400 μL of 80% methanol aqueous solution was added. The mixture was vortexed and centrifuged. The supernatant was then diluted with mass spectrometry-grade water to achieve a final methanol concentration of 53%. After centrifugation, the supernatant was collected for LC-MS analysis. A 53% methanol aqueous solution was used as a blank control. Additionally, equal volumes from each experimental sample were pooled and mixed to create a quality control (QC) sample, which was used to monitor the entire analytical process, ensuring instrument stability and repeatability.

#### 2.6.2 Chromatographic condition

The chromatographic analysis was performed using a Hypersil Gold (C18) column with the temperature of 40 C and a flow rate of 0.2 mL/min. For the positive ion mode, the mobile phase consisted of 0.1% formic acid (mobile phase A) and methanol (mobile phase B). For the negative ion mode, the mobile phase consisted of 5 mM ammonium acetate with a pH 9.0 (mobile phase A) and methanol (mobile phase B).

#### 2.6.3 Mass spectrometry condition

Mass spectrometric detection was performed with a mass-to-charge ratio (m/z) range of 100–1500, using an ESI source with the following parameters: spray voltage at 3.5 kV; sheath gas flow rate at 35 psi; auxiliary gas flow rate at 10 L/min; capillary temperature at 320 C; S-lens RF level at 60; auxiliary gas heater temperature at 350 C; polarity set to both positive and negative mode; MS/MS secondary scanning was conducted using data-dependent scans.

#### 2.6.4 Data preprocessing and metabolite identification

The raw data files (.raw) were imported into Compound Discoverer 3.1 (CD3.1) software for initial filtration and simple screening. Peak alignment and peak extraction were performed across different samples, followed by quantification of peak areas and integration of target ions. Molecular formulas were predicted using molecular ion peaks and fragment ions, which were then compared against mzCloud (https://www.mzcloud.org/), mzVault, and Masslist databases. The data were normalized using the formula: raw quantification value of the sample / (total quantification value of metabolites in the sample / total quantification value of metabolites in QC sample 1), to obtain relative peak areas. Compounds with a coefficient of variation (CV) greater than 30% in the QC sample were excluded, resulting in the final identification and relative quantification of metabolites. Data processing was conducted using the Linux operating system (CentOS 6.6) and software tools R and Python.

### 2.7 16S rRNA gene sequencing

Genomic DNA was extracted from fecal samples using cetyltrimethylammonium bromide (CTAB). The purity and concentration of the extracted DNA were assessed using 0.8% agarose gel electrophoresis. The bacterial 16S rDNA genes V3–V4 regions were amplified using the forward primer 341F (CCTAYGGGRBGCASCAG) and the reverse primer 806R (GGACTACNNGGGTATCTAAT). Specific primers with barcodes and Phusion^®^ High-Fidelity PCR Master Mix with GC Buffer were employed to ensure the accuracy and efficiency of amplification. PCR products were pooled in equal amounts based on their concentrations, and the amplification results were subjected to 2% agarose gel electrophoresis. The target bands were then recovered using a gel extraction kit. Negative extraction controls and blank PCR controls were included in each run, and no amplification products were detected. A sequencing library was constructed using the TruSeq^®^ DNA PCR-Free Sample Preparation Kit (Illumina). After quantification with Qubit and Q-PCR, libraries that met the quality criteria were sequenced using the NovaSeq 6000 system to target the 16S rRNA V3–V4 regions.

### 2.8 Bioinformatics analysis

Raw reads were processed using cutadapt (v3.3) to remove primers ( ≤ 4 mismatches), assembled by FLASH (v1.2.11) with a minimum overlap of 10 bp and maximum mismatch density of 0.2, and quality-filtered using fastp (v0.23.1) with a minimum average Q-score of 19 and a low-quality ratio threshold of 15%; reads < 20 bp were discarded. Operational Taxonomic Units (OTUs) were clustered at 97% similarity using Uparse (v7.0.1001) and annotated against the SSUrRNA database via Mothur. Taxonomic assignments were interpreted at the genus level, as V3–V4 amplicons (~460 bp) do not reliably resolve species.

For diversity analysis, samples were rarefied to the minimum sequencing depth (rarefaction curves plateaued). Median read count per sample was 118,837 reads (range: 46,772–135,589), and rarefaction curves reached a plateau. The rarefied OTU table was used for alpha and beta diversity, ordination, and LEfSe analyses to identify differential taxa between groups.

For metabolomics results, the identified metabolites were annotated using KEGG database (https://www.genome.jp/kegg/pathway.html).

### 2.9 Statistical analysis

Statistical analysis was performed using SPSS software version 22.0. Continuous variables were first tested for normality with the Shapiro–Wilk. Variables with a normal distribution were expressed as mean ± standard deviation ($\bar{x}\pm$ s), while those with a non-normal distribution were expressed as the median (first quartile, third quartile). For comparisons among the three groups, if the data are normally distributed, one-way ANOVA was used. The F-test was applied for homogeneity of variances, with pairwise comparisons between groups conducted using the LSD test. If the variance was not homogeneous, the Welch test was used, and pairwise comparisons were conducted using Tamhane's test. Nonparametric tests were employed for data that did not follow a normal distribution. For comparisons between the two groups, an independent samples *t*-test was used for normally distributed data. Categorical data were analyzed using the chi-square test. A *p*-value < 0.05 was considered statistically significant. Correlation analyses between differential gut microbiota and differential metabolites were performed using Spearman's rank correlation in R software (version 4.5.0) and visualized as a heat map. *P*-values from differential genera (independent samples t-test), differential metabolites (selected by VIP ≥ 1, FC > 1.5, *P* < 0.05), and Spearman correlations were corrected for multiple testing using the Benjamini–Hochberg false discovery rate (FDR) method. Adjusted q-values are reported, and *q* < 0.05 was considered statistically significant.

## 3 Results

### 3.1 Characteristics of study participants

A total of 110 participants were included in the study: 40 in Group I (sleep disorder group), 40 in Group N (non-sleep disorder group), and 30 in Group ZC (healthy control group). In Group I, there were 20 males and 20 females, aged 34–78 years, with 0.25–26 years of diabetes duration. Group N included 27 males and 13 females, aged 31–77 years, with 0.25–20 years of diabetes duration. Group ZC comprised 20 males and 10 females, aged 39–76 years. There were no statistically significant differences in gender, age, height, or weight among the three groups (*p* > 0.05, Table 1). Additionally, no significant difference was found between group I and group N in the use of glucose-lowering medications (*p* > 0.05, Supplementary Table S1). However, there were significant differences in waist circumference among the three groups (*P* < 0.05), and pairwise comparisons revealed no significant difference between Groups I and N (*p* > 0.05). The waist circumference of both diabetic groups was significantly higher than that of Group ZC (*p* < 0.05). It may relate to abnormal fat metabolism and accumulation in diabetic patients. There was no significant difference in the duration of diabetes between Groups I and N (*p* = 0.17). All participants collected samples as required and completed the entire trial without any dropouts.

**Table 1:** Clinical characteristics of the participants (*n* = 110).

Group	Number	Gender (%)		Age (year)	Height (cm)	Weight (kg)	Waistline (cm)	Course of diabetes (year)	PSQI score
		Male	Female						
I	40	20 (50.0)	20 (50.0)	58.55 ± 10.04	164.43 ± 6.71	70.11 ± 10.78	91.8 ± 8.92^a^	10.53 ± 6.76	18.53 ± 1.77^a, b^
N	40	27 (67.5)	13 (32.5)	55.8 ± 11.24	166.63 ± 6.74	70.55 ± 10.85	90.85 ± 10.17^a^	8.56 ± 6.10	2.53 ± 1.95
ZC	30	20 (66.6)	10 (33.3)	54.53 ± 7.80	166.63 ± 7.19	70.67 ± 9.92	85.07 ± 8.84	-	2.80 ± 1.63
χ2/F/H/t		3.147^①^		1.528^②^	1.315^②^	0.464^③^	6.848^④^	1.37^④^	138.21^②^
p		0.207		0.222	0.273	0.793	0.033	0.17	< 0.001

: χ2 values. : F value. : H value. : t value. a: *p* < 0.05 compared with the Group ZC. b: *p* < 0.05 compared with Group N. Data are presented as the mean ± SD. *n*: sample size; -: no data; Group I: sleep disorder group; Group N: non-sleep disorder group; Group ZC: healthy control group.

### 3.2 Comparison of blood glucose and lipid levels among the groups

There were significant statistical differences in FPG, 1hPBG, 2hPBG and HDL-C levels among the three groups (*P* < 0.05, Table 2). Specifically, the FPG, 1hPBG, and 2hPBG levels were significantly higher in Groups I and N compared to Group ZC. Additionally, the levels of HDL-C was lower in Group N compared to Group ZC. For the remaining lipid and glucose levels, the differences between groups were not statistically significant (*P* > 0.05). There were no significant differences in blood glucose and lipid levels between Group I and Group N (*P* > 0.05).

**Table 2:** Comparison of blood glucose and lipid levels among different groups (*n* = 110).

Indicator	Group I	Group N	Group ZC	F/H	*P*
FPG (mmol/L)	8.47 (7.15, 10.91)^a^	8.87 (6.81, 11.14)^a^	5.15 (4.94, 5.39)	46.54②	< 0.01
1hPBG (mmol/L)	14.98 ± 4.00^a^	16.11 ± 4.32^a^	9.99 ± 0.89	3.39^①^	0.04
2hPBG (mmol/L)	14.73 (12.58, 18.27)^a^	14.96 (12.29, 19.36)^a^	5.58 (4.78, 9.53)	8.06^②^	< 0.01
FIns (pmol/L)	51.07 (33.48, 64.88)	50.31 (27.12, 92.19)	45.47 (31.59, 61.15)	0.67^②^	0.71
FC-P (nmol/L)	0.63 (0.50, 0.78)	0.66 (0.52, 1.00)	0.96 (0.66, 1.16)	3.11^②^	0.21
TG (mmol/L)	1.34 (1.07, 2.00)	1.39 (1.04, 1.88)	1.37 (0.81, 1.91)	0.58^②^	0.75
TC (mmol/L)	4.18 (3.51, 4.64)	4.55 (3.17, 4.97)	4.70 (4.07, 5.09)	2.83^②^	0.24
UA (μmol/L)	263.20 (208.90, 307.15)	265.55 (230.88, 321.83)	246.70 (202.35, 305.85)	0.80^②^	0.67
HDL-C (mmol/L)	1.14 (0.95, 1.57)	1.01 (0.86,1.14)^a^	1.47 (1.26,1.69)	17.61^②^	< 0.01
LDL-C (mmol/L)	2.16 (1.76, 2.76)	2.06 (1.38, 2.93)	2.54 (1.85, 3.05)	2.56^②^	0.28
Lp(a) (mg/L)	108.80 (65.50, 192.18)	88.45 (47.93, 205.03)	159.35 (96.35, 488.70)	5.36^②^	0.07
ApoA1 (g/L)	1.27 (1.11, 1.52)	1.16 (1.07, 1.32)	1.23 (1.13, 1.33)	5.65^②^	0.06
ApoB (g/L)	0.87 ± 0.23	0.87 ± 0.23	0.97 ± 0.17	2.27^①^	0.11
ApoE (mg/L)	43.20 (33.73, 48.60)	38.30 (29.05, 52.08)	45.95 (39.78, 53.43)	3.87^②^	0.14

: F value. : H value. a: *p* < 0.05 compared with the Group ZC. Data are presented as the mean ± SD. FPG: fasting plasma glucose; 1hPBG, 1-hour postprandial blood glucose; 2hPBG, 2-hour postprandial blood glucose; FIns, fasting insulin; FC-P, fasting C-peptide; ApoA1, apolipoprotein A1; TG, triglycerides; TC, total cholesterol; UA, uric acid; HDL-C, high-density lipoprotein cholesterol; LDL-C, low-density lipoprotein cholesterol; Lp(a), lipoprotein(a); ApoB, apolipoprotein B; ApoE, apolipoprotein E.

### 3.3 Gut microbiota diversity and richness analysis

Firstly, the Shannon-Wiener curve was used to assess the depth of sequencing in each group. The Shannon curves tended toward flatness, indicating that the sequencing data were sufficient to reflect most of the microbial information in the samples, making it suitable for subsequent analysis (Figure 1A). Furthermore, the Good's coverage index for all three groups exceeded 0.9998, suggesting high microbial coverage that accurately reflects the true microbial composition of the samples.

**Figure 1:**
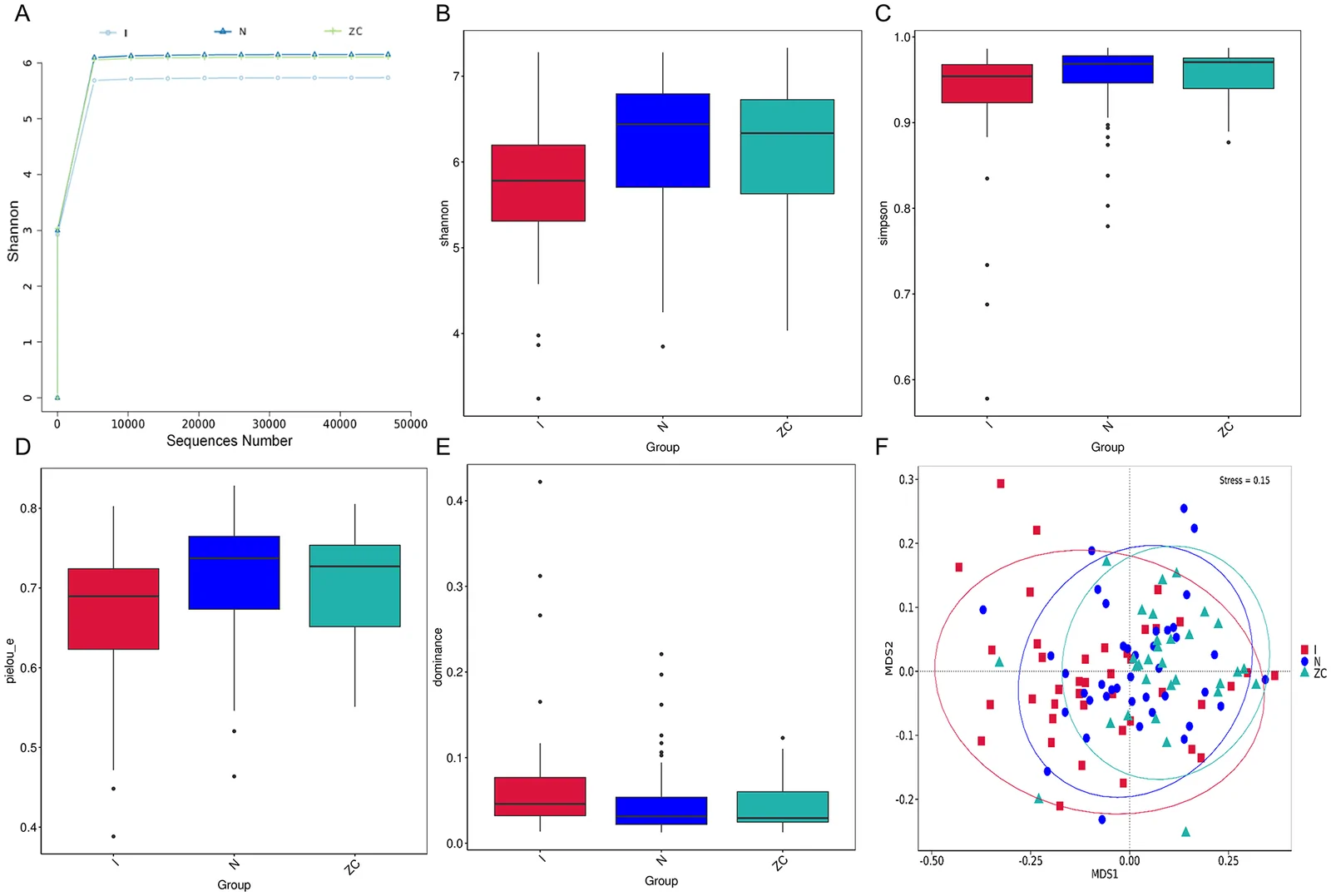
Comparison of gut microbiota diversity and richness among groups. **(A)** Shannon-Wiener curve. **(B-E)** Shannon index, Simpson index, Pielou's index, and Dominance index for alpha diversity. **(F)** Non-metric multidimensional scaling (NMDS) two-dimensional plot for beta diversity (*n* = 110).

Next, alpha diversity analysis revealed significant differences between the diabetic patients with and without sleep disorders. Group I exhibited lower taxa diversity compared to Group N, as indicated by the Shannon and Simpson indices (*P* = 0.01 and *P* = 0.03, respectively; Figures 1B, C). The Pielou's evenness index was also lower in Group I than in Group N (*P* < 0.01; Figure 1D), and the Dominance index was higher in Group I (*P* = 0.01; Figure 1E), collectively suggesting a more uneven distribution of taxa and lower relative abundance within Group I.

Further, beta diversity analysis showed a non-metric multidimensional scaling (NMDS) stress value was 0.15, indicating that the two-dimensional plot adequately represents the data distribution (Figure 1F). The NMDS plot showed substantial overlap between the microbial communities of Groups I and N, suggesting limited separation between the two groups. PERMANOVA based on Weighted UniFrac distances further revealed a small but statistically significant difference in overall microbial community structure between Groups I and N (*R*^2^ = 0.0306, *P* = 0.041). Pairwise comparisons also showed significant differences between Group I and Group ZC (*R*^2^ = 0.1112, *P* = 0.001) and between Group N and Group ZC (*R*^2^ = 0.0457, *P* = 0.009).

### 3.4 Analysis of differential taxa among groups

LEfSe analysis indicated that each group possessed dominant bacterial taxa, with a total of 17 bacterial taxa showing significant differences in the abundance among groups (*P* < 0.05). Specifically, Group I had a higher abundance of seven gut microbiota taxa, including *Selenomonadaceae, Pseudomonadaceae, Pseudomonas, Megamonas, Pseudomonadales, Gammaproteobacteria*, and *Proteobacteria* compared to Groups N and ZC, marking them the dominant taxa in Group I (*P* = 0.0002, *P* = 0.0000, *P* = 0.0000, *P* = 0.0000, *P* = 0.001, *P* = 0.0001, *P* = 0.0001, respectively). Conversely, in Group N, *Veillonellaceae, Escherichia_Shigella*, and *Veillonellales_Selenomonadales* were more prevalent than in Groups I and ZC, indicating them as dominant taxa in Group N (*P* = 0.02, *P* = 0.0000, *P* = 0.01, respectively). Meanwhile, in Group ZC, *Bacteroidota, Bacteroidaceae, Prevotellaceae, Prevotella, Bacteroidales, Bacteroides*, and *Bacteroidia* exhibited a greater abundance compared to Groups I and N, establishing them as the dominant taxa (*P* = 0.0002, *P* = 0.03, *P* = 0.003, *P* = 0.005, *P* = 0.0002, *P* = 0.03, *P* = 0.0002, respectively; Figure 2A).

**Figure 2:**
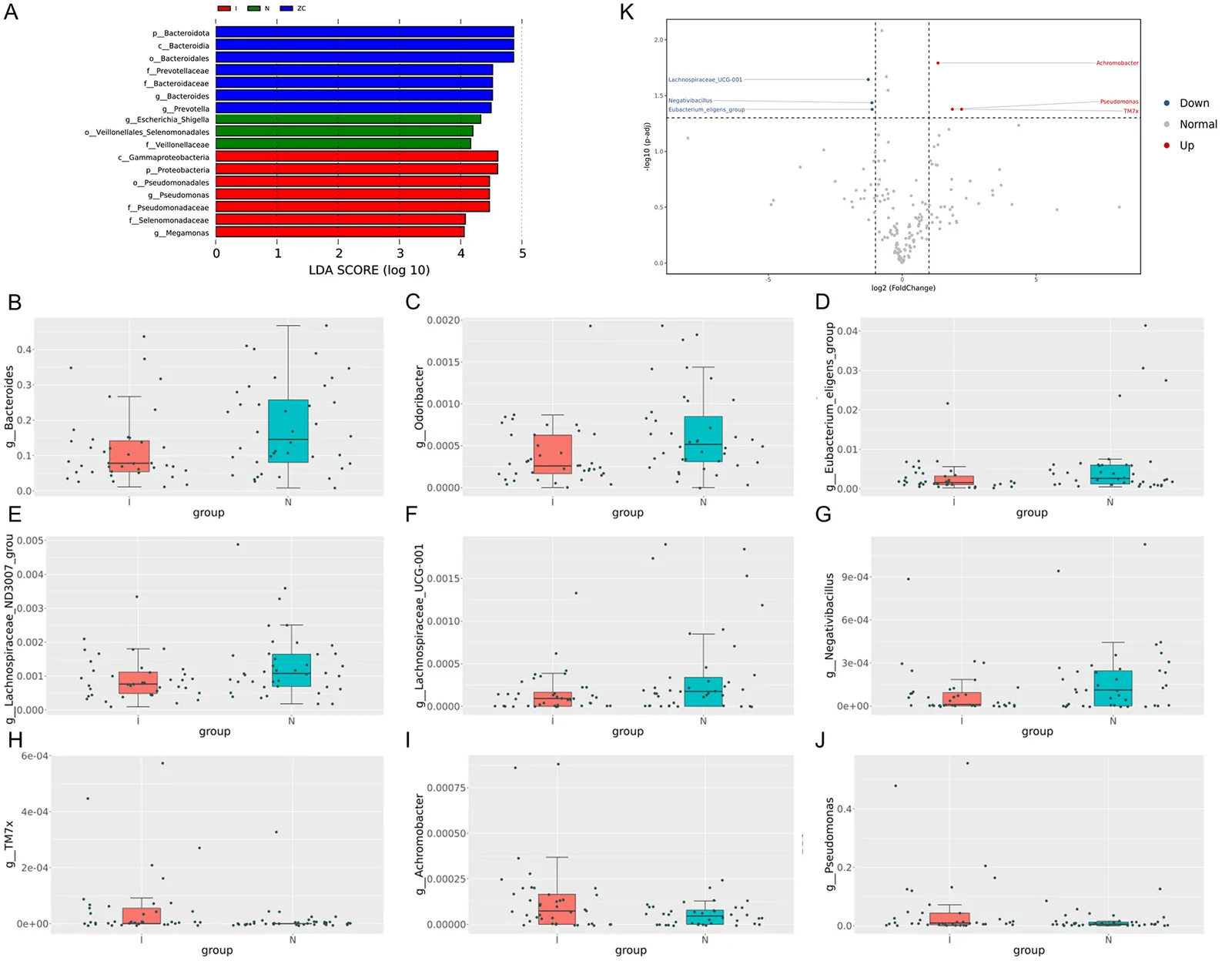
Differential taxa among groups. **(A)** Histogram of linear discriminant analysis (LDA) score distribution highlighting bacterial taxa with significant differences, identified using an LDA score (log10) >4.0 (*n* = 110). **(B-J)** Box plots of different genera, including *Bacteroides, Odoribacter, Eubacterium_eligens_group, Lachnospiraceae_ND3007, Lachnospiraceae_UCG-001, Negativibacillus, TM7x, Achromobacter*, and *Pseudomonas* (*n* = 80). **(K)** Volcano plot of differential genera (*n* = 80).

Further analysis at the genus level identified nominal differences between Groups I and N. T-tests and box plot analysis showed that nine genera exhibited nominal differences in abundance between the two groups (*P* < 0.05, Figures 2B-J). Specifically, the abundance of *TM7x, Achromobacter*, and *Pseudomonas* showed higher abundance in Group I compared to Group N (*P* = 0.04, *P* = 0.02, *P* = 0.04, respectively). In Group N, abundance of *Bacteroides, Odoribacter, Eubacterium_eligens_group, Lachnospiraceae_ND3007, Lachnospiraceae_UCG-001*, and *Negativibacillus* were significantly higher compared to Group I (*P* = 0.02, *P* = 0.008, *P* = 0.04, *P* = 0.03, *P* = 0.02, *P* = 0.04, respectively). Similarly, volcano plot analysis of differential genera between Groups I and N highlighted nominal increases in *TM7x, Achromobacter*, and *Pseudomonas* in Group I (Supplementary Table S2). In contrast, lower relative abundance were observed in *Eubacterium_eligens_group, Lachnospiraceae_UCG-001*, and *Negativibacillus*. These findings suggested that the six differential genera identified may represent candidate microbial features in the analysis of sleep disorders among diabetic patients (Figure 2K). After Benjamini–Hochberg correction across the 178 genera tested, none of the nine genera remained significant (adjusted *q* = 0.71 for all nine genera), indicating that these genus-level differences are nominal and should be interpreted as exploratory rather than confirmed.

To assess whether the genus-level differences remained after adjusting for clinical confounders, a MaAsLin2 multivariate model was fitted with sleep status as the primary effect and age, sex, BMI, diabetes duration, and metformin use as covariates. After correction for multiple testing, no genus reached statistical significance (minimum *q* = 0.754). At the nominal level (*P* < 0.05), *TM7x* (coefficient +0.93, *P* = 0.005) and *Eubacterium_eligens_group* (coefficient −0.95, *P* = 0.009) retained associations in the same direction as the univariate analysis, whereas *Achromobacter* (*P* = 0.085), *Pseudomonas* (*P* = 0.171), and *Negativibacillus* (*P* = 0.050) did not meet the nominal significance threshold, and *Lachnospiraceae_UCG-001* was excluded by the 10% prevalence filter. These results indicate that the genus-level associations were attenuated after covariate adjustment and did not remain significant after multiple-testing correction. Therefore, they should be regarded as exploratory signals rather than confirmed disease-associated signatures.

### 3.5 Metabolite analysis and identification of differential pathways

The Pearson correlation index between the QC samples was calculated based on the relative quantitation of the metabolites, showing an R^2^ close to 1. This indicates a high correlation among QC samples, suggesting the accuracy and reliability of the experimental data, as well as the stability of the instrument (Figure 3A). Partial Least Squares Discriminant Analysis (PLS-DA) was employed as a supervised statistical method to assess the data, where R^2^ evaluates the explanatory power of the PLS-DA model, and Q^2^ assesses the model's predictive ability. The PLS-DA results revealed that significant differences were observed between Group I and Group ZC, as well as between Group N and Group ZC. Although there was some overlap between Group I and Group N, distinct trends were still present between these two groups (Figure 3B).

**Figure 3:**
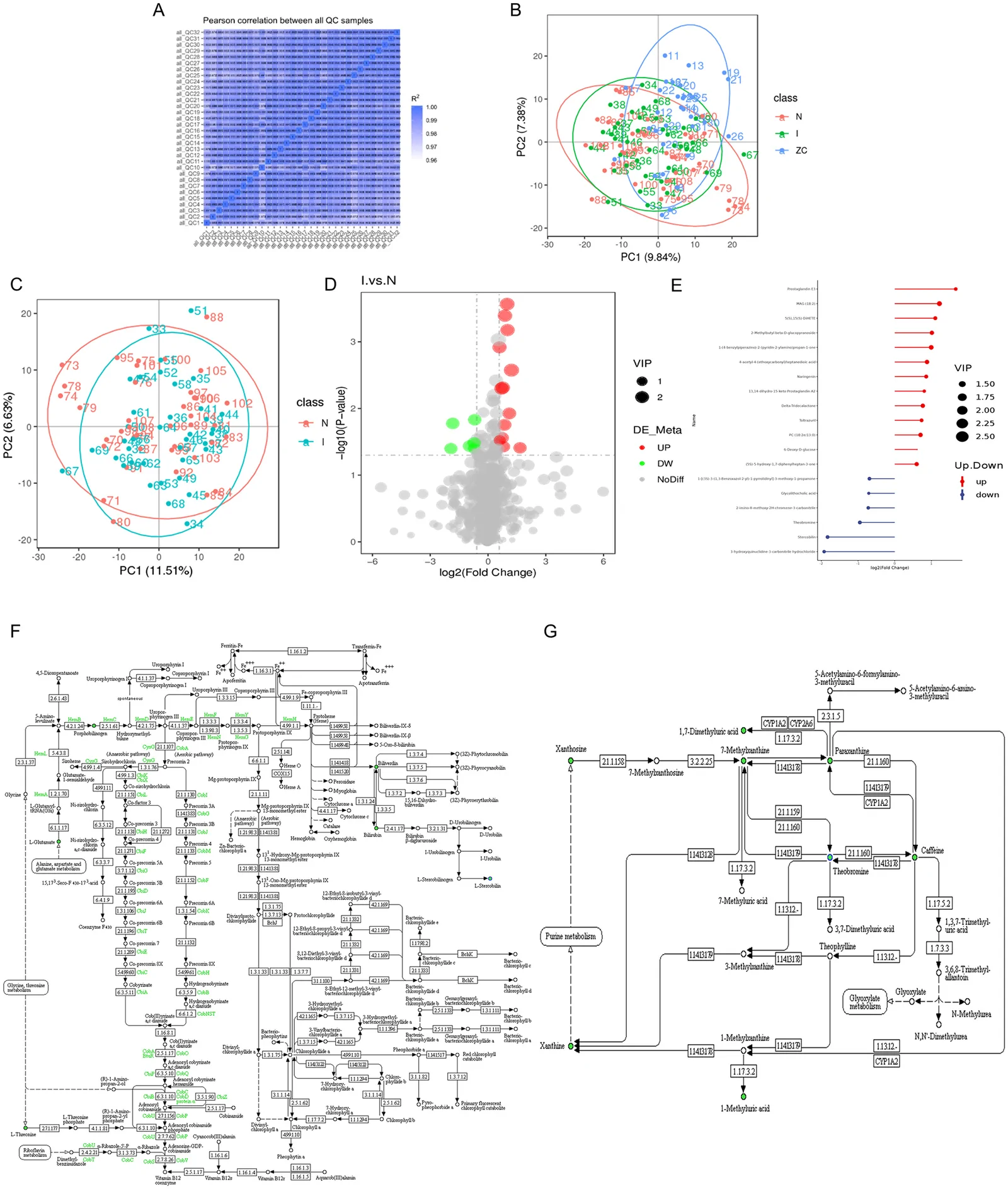
Metabolomics analysis of Group I and Group N. **(A)** Quality control results. **(B)** Partial least squares discriminant analysis (PLS-DA) score scatter plot among the three groups (*n* = 110). **(C-G)** Analyses comparing Group I and Group N (*n* = 80): **(C)** PCA score plot. **(D)** Volcano plot of differential metabolites. **(E)** Stem plot of differential metabolites. **(F-G)** Kyoto Encyclopedia of Genes and Genomes (KEGG) pathway enrichment analysis showing two candidate pathways: porphyrin and chlorophyll metabolism and caffeine metabolism (*P* = 0.12 and *P* = 0.14, respectively).

#### 3.5.1 Impact of sleep disorders on metabolites in diabetic patients (Group I vs. Group N)

To further explore the impact of sleep disorders on serum metabolites, the differences in serum metabolites and metabolic pathways between Group I and Group N were compared. Unsupervised PCA analysis revealed that although Group I and Group N samples partially overlapped in the principal component space, a tendency toward separation was still discernible (Figure 3C). Using VIP ≥ 1, FC > 1.5, and *P* < 0.05 as criteria, 17 differential metabolites were identified between Group I and Group N (Table 3). Among these, 14 metabolites were classified as MSI Level 2, while the remaining 3 metabolites were assigned to MSI Level 3. Two additional metabolites initially flagged as differential—toltrazuril and 1-(4-benzylpiperazino)-2-(pyridin-2-ylamino)propan-1-one—were excluded from Table 3 and treated as putative annotations due to their chemical implausibility as endogenous human serum metabolites. The volcano and stem plots showed that compared to Group N, 11 metabolites were upregulated, whereas 6 were downregulated in Group I (Figures 3D, E). None of these metabolites remained significant after Benjamini–Hochberg correction (minimum *q* = 0.197), indicating that these differences are nominal and exploratory.

**Table 3:** Differential metabolite information between Group I and Group N (*n* = 80).

Number	Name	Formula	VIP	*P* value	*q* value (BH)	FC	Trend	ROC	MSI Level
1	4-acetyl-4-(ethoxycarbonyl)heptanedioic acid	C_12_H_18_O_7_	2.27	< 0.01	0.197	1.84	↑	0.71	2
2	2-Methylbutyl beta-D-glucopyranoside	C_11_H_22_O_6_	2.21	< 0.01	0.216	2.02	↑	0.70	2
3	(5S)-5-hydroxy-1,7-diphenylheptan-3-one2.59	C_19_H_22_O_2_	2.15	< 0.01	0.235	1.51	↑	0.67	2
4	MAG (18:2)	C_21_H_38_O_4_	2.59	< 0.01	0.431	2.32	↑	0.69	2
5	Delta-Tridecalactone	C_13_ H_24_ O_2_	2.23	< 0.01	0.541	1.70	↑	0.68	2
6	PC (18:2e/13:0)	C_39_H_76_NO_7_P	2.26	< 0.01	0.541	1.63	↑	0.65	2
7	5(S),15(S)-DiHETE	C_20_H_32_O_4_	2.00	0.01	0.768	2.15	↑	0.65	2
8	1-[(3S)-3-(1,3-Benzoxazol-2-yl)-1-pyrrolidinyl]-3-methoxy-1-propanone	C_15_H_18_N_2_O_3_	1.89	0.01	0.813	0.62	↓	0.66	3
9	Stercobilin	C_33_H_46_N_4_O_6_	1.89	0.02	0.813	0.28	↓	0.65	2
10	Naringenin	C_15_H_12_O_5_	1.99	0.02	0.813	1.81	↑	0.65	2
11	6-Deoxy-D-glucose	C_6_H_12_O_5_	1.32	0.03	0.813	1.52	↑	0.64	2
12	2-imino-8-methoxy-2H-chromene-3-carbonitrile	C_11_H_8_N_2_O_2_	1.80	0.03	0.813	0.61	↓	0.63	3
13	Theobromine	C_7_H_8_N_4_O_2_	1.95	0.04	0.813	0.52	↓	0.62	2
14	13,14-dihydro-15-keto Prostaglandin A2	C_20_H_30_O_4_	1.74	0.04	0.813	1.75	↑	0.64	2
15	Prostaglandin E3	C_20_H_30_O_5_	1.83	0.04	0.813	3.17	↑	0.60	2
16	Glycolithocholic acid	C_26_H_43_NO_4_	1.64	0.04	0.813	0.61	↓	0.65	2
17	3-hydroxyquinuclidine-3-carbonitrile hydrochloride	C_8_H_12_N_2_O	1.77	0.04	0.813	0.26	↓	0.65	3

The trend of change is based on the baseline of Group N, indicating an increase or decrease in Group I relative to Group N. VIP, variable importance in projection; FC, fold change; ROC, receiver operating characteristic; MSI, metabolomics standards initiative. MSI Level 2, putatively annotated compounds (MS/MS spectral match); MSI Level 3, putatively characterized compound classes (MS1 data only).

The 17 nominally differential metabolites identified between Group I and Group N were further analyzed for metabolic pathways enrichment analysis. Stercobilin and theobromine were mapped to porphyrin and chlorophyll metabolism and caffeine metabolism, respectively (Figures 3F-G). However, neither pathway enrichment reached statistical significance (*P* = 0.12 and *P* = 0.14, respectively).

#### 3.5.2 Pentose phosphate pathway was identified as the differential metabolic pathway between Group I and Group ZC

The results showed a clear separation between Group I and Group ZC, indicating significant differences between these two groups (Figure 4A). The model parameters were R^2^Y = 0.90 and *Q*^2^ = 0.74, suggesting good explanatory and predictive capabilities of the model. The regression line plot showed that *R*^2^ was greater than *Q*^2^, and the *Q*^2^ regression line intersected the Y-axis below zero, indicating that the model was not overfitted and could accurately describe the samples, providing a basis for screening candidate features (Figure 4B). Using criteria of VIP ≥ 1, FC > 1.5, and *P* < 0.05, 114 differential metabolites were identified between Group I and Group ZC (Figures 4C, D), and their related metabolic pathways were further analyzed on the MetaboAnalyst 5.0 platform, resulting in 22 metabolic pathways being identified. Among these, the pentose phosphate pathway was identified as a candidate pathway (*P* < 0.05, Figure 4E), involving two differential metabolites: Gluconolactone and D-gluconic acid. Research indicates that the pentose phosphate pathway is closely related to the development and progression of diabetes, participating in the regulation of blood sugar (Zhu et al., 2026). Disruptions in this pathway can lead to abnormal synthesis and breakdown of glucose, resulting in glucose imbalances and an increased risk of diabetes. However, as this comparison reflects diabetes-associated alterations rather than sleep-disorder-specific effects, these results should be interpreted as background context for the diabetic state.

**Figure 4:**
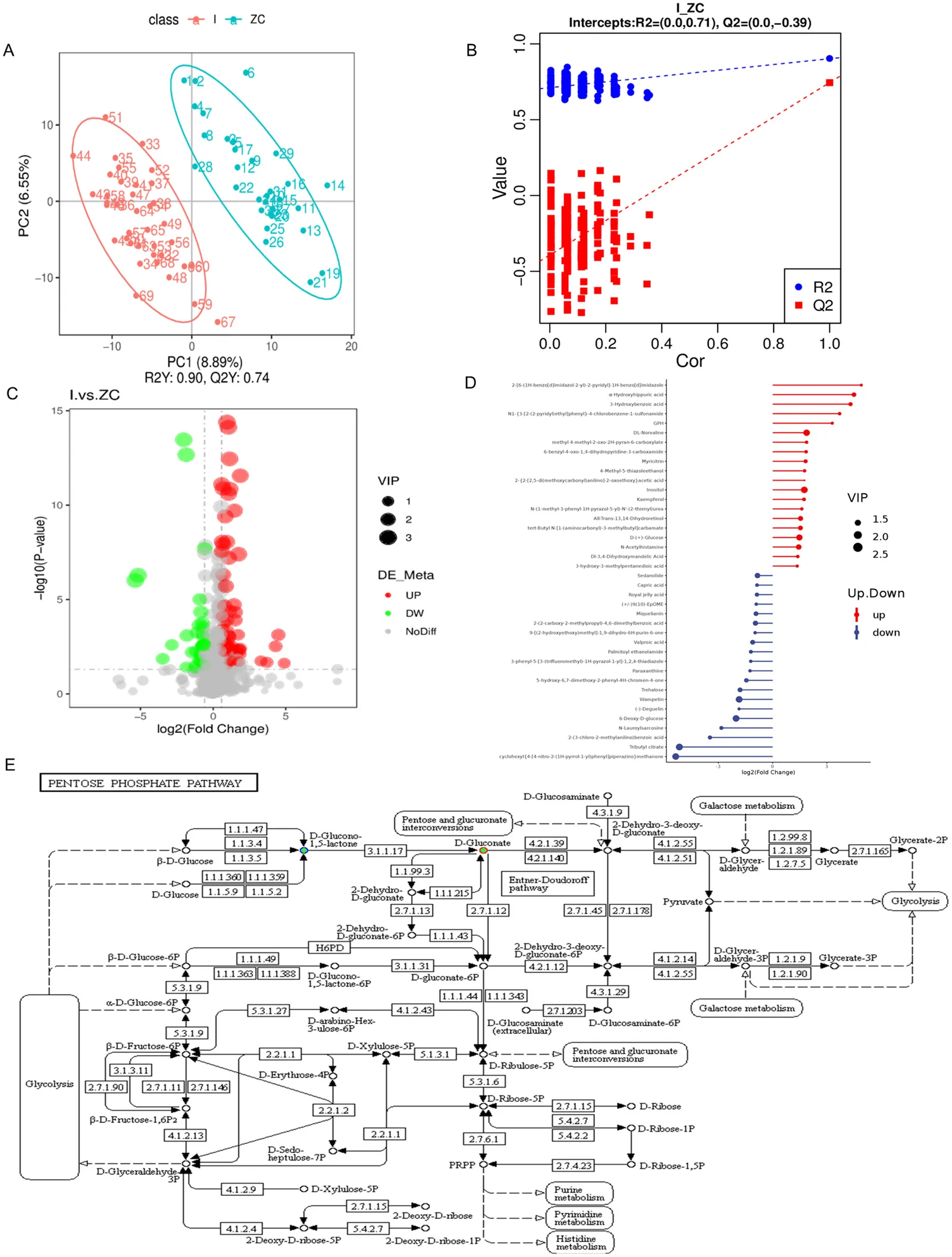
Metabolomics analysis of Group I and Group ZC. **(A)** Partial least squares discriminant analysis (PLS-DA) score plot. **(B)** Permutation test of the PLS-DA model. **(C)** Volcano plot of differential metabolites. **(D)** Stem plot of the top 40 differential metabolites. **(E)** Kyoto Encyclopedia of Genes and Genomes (KEGG) pathway enrichment analysis showing the pentose phosphate pathway (*n* = 70).

#### 3.5.3 Differential metabolomics analysis of bile acid and cholesterol metabolic pathways between Group N and Group ZC

To characterize the metabolic differences between Group N and Group ZC, untargeted metabolomics was performed based on an PLS-DA model (R^2^Y = 0.90 and *Q*^2^ = 0.74, with no overfitting, Figures 5A, B). Using the criteria of VIP ≥ 1, FC > 1.5, and *P* < 0.05, a total of 106 differential metabolites were identified, encompassing carbohydrates, amino acids, organic acids, and lipids (Supplementary Table S3, Figures 5C, D). KEGG pathway enrichment analysis identified 22 significantly enriched metabolic pathways, with the top three candidate pathways being primary bile acid biosynthesis (*P* = 0.0025), bile secretion (*P* = 0.0025), and cholesterol metabolism (*P* = 0.0424, Figures 5E-G). In conclusion, Group N and Group ZC showed differences in bile acid synthesis, bile secretion, and cholesterol metabolism. These findings are presented primarily as background context for diabetes-associated metabolic alterations rather than sleep-disorder-specific changes.

**Figure 5:**
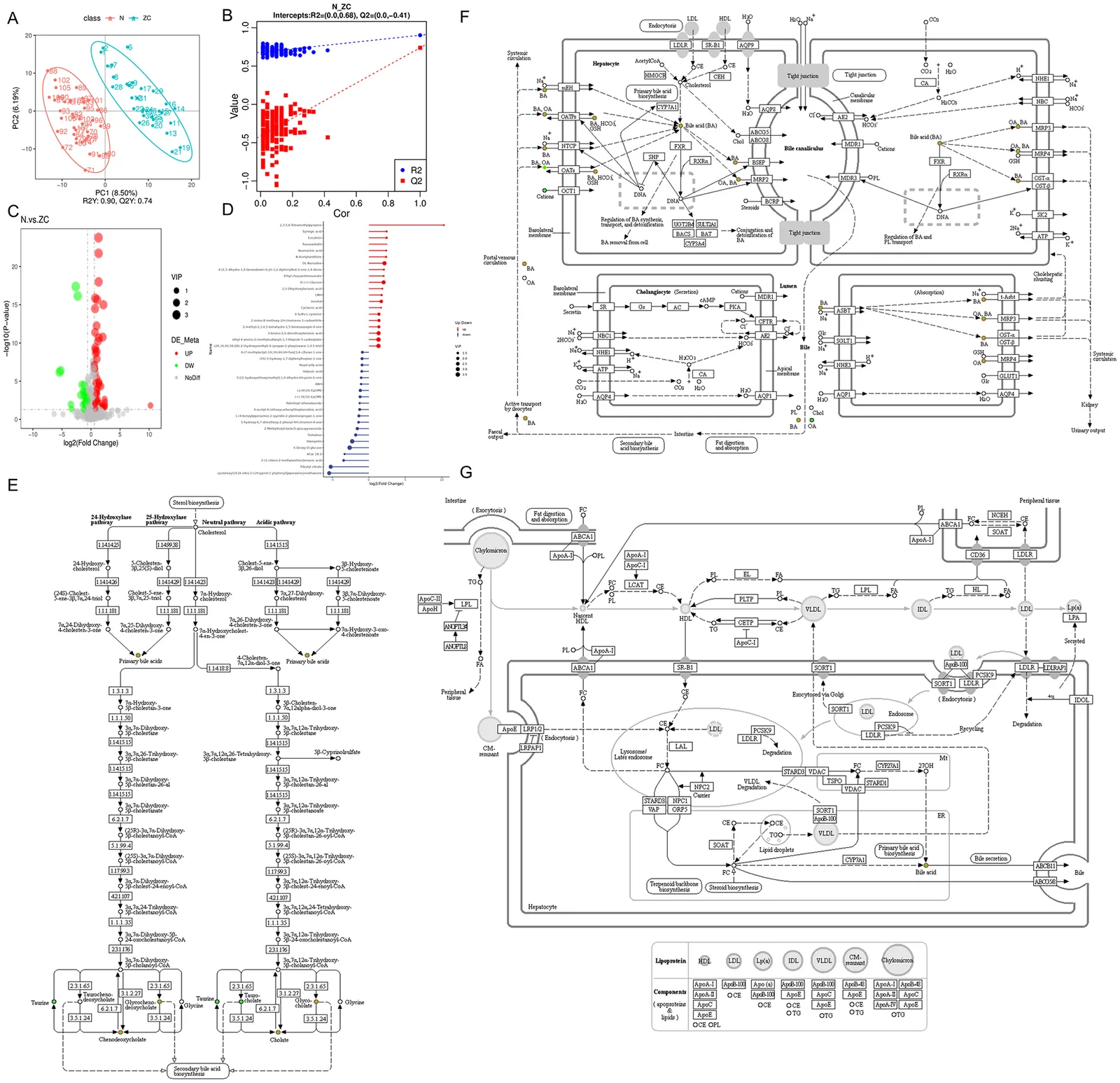
Metabolomics analysis of Group N and Group ZC. **(A)** Partial least squares discriminant analysis (PLS-DA) score plot. **(B)** Permutation test of the PLS-DA model. **(C)** Volcano plot of differential metabolites. **(D)** Stem plot of the top 40 differential metabolites. **(E-G)** KEGG pathway enrichment analysis of the differential metabolites, showing the top three significantly enriched pathways: primary bile acid biosynthesis **(E)**, bile secretion **(F)**, and cholesterol metabolism **(G)** (*n* = 70).

### 3.6 Microbe-metabolite associations

A total of 10 correlations with unadjusted *P* < 0.05 were detected between 9 differential gut microbiota and 17 differential serum metabolites. Of these, seven pairs showed positive correlations and three pairs showed negative correlations. Stercobilin was positively correlated with *Lachnospiraceae_UCG-001* (*R* = 0.381, *P* < 0.001, *Q* = 0.095), *Lachnospiraceae_ND3007* (*R* = 0.354, *P* = 0.002, *Q* = 0.121), and *Odoribacter* (*R* = 0.280, *P* = 0.01, *Q* = 0.301; Figure 6).

**Figure 6:**
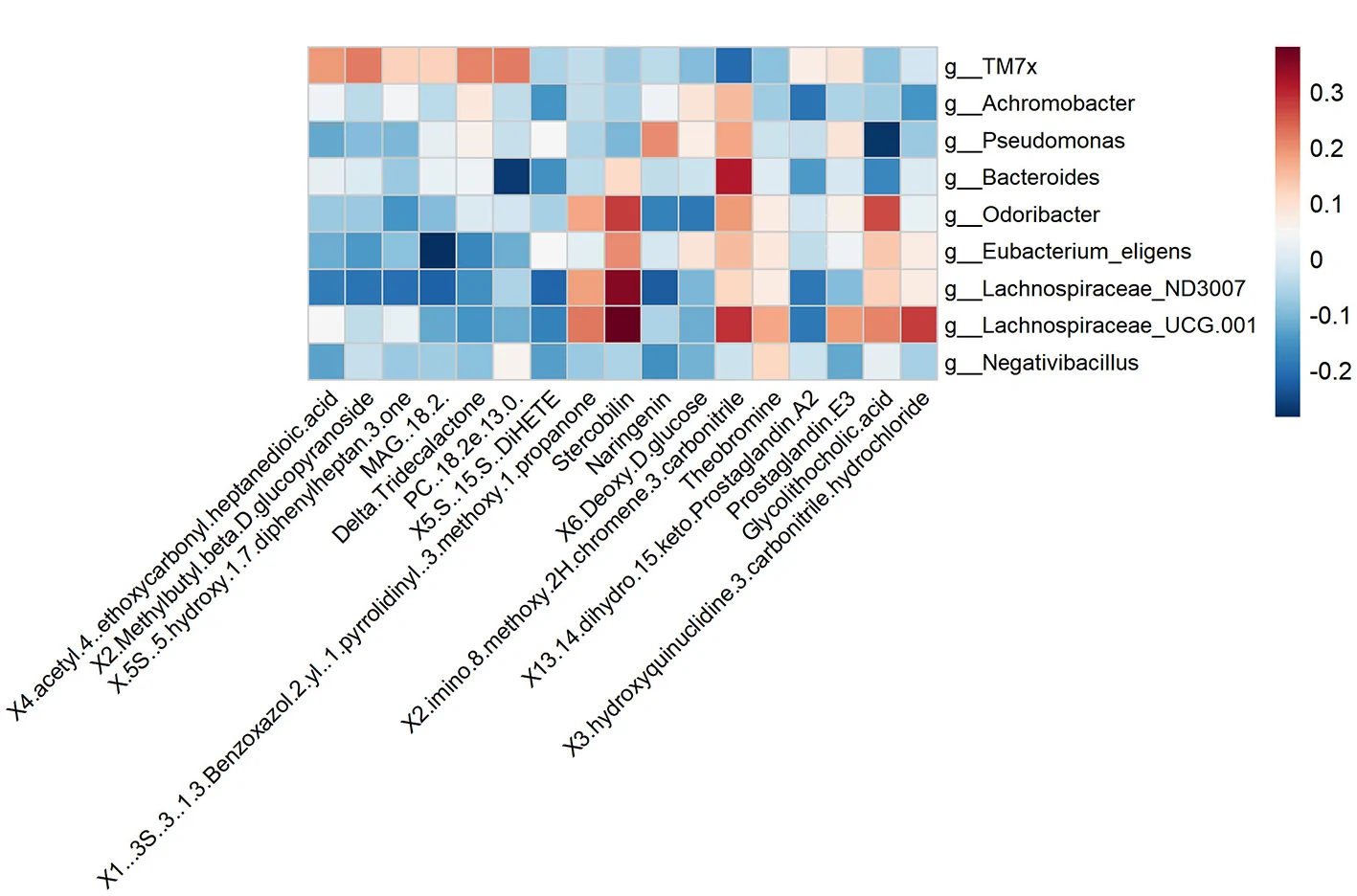
Heat map of Spearman correlation analysis between gut microbiota and metabolites (*n* = 80).

## 4 Discussion

Sleep disorders are common comorbidities in patients with T2DM and have been increasingly recognized as important factors affecting metabolic health and quality of life. Therefore, exploring the pathological mechanisms of T2DM with sleep disorders is of great importance. Utilizing comprehensive 16S rRNA gene sequencing and metabolomic analysis, this study discovered a notable decrease in gut microbiota diversity and evenness in T2DM patients with sleep disorders than those without. Variations in the taxonomic composition and diversity of the gut microbiota were also observed between the two groups. And several differential microbial taxa were identified. Furthermore, metabolomic analysis identified 17 differential metabolites, enriched in porphyrin and chlorophyll metabolism and caffeine metabolism pathways.

Analysis of gut microbiota across groups revealed that both diabetic groups, regardless of sleep disorder status, showed significantly decreased abundance of Bacteroidota, Bacteroidaceae, *Prevotella*ceae, *Prevotella*, Bacteroidales, *Bacteroides*, and Bacteroidia. In particular, *Bacteroides* and *Prevotella*, well-known anaerobes involved in carbohydrate metabolism and short-chain fatty acid production, were markedly depleted, consistent with several previous studies (Lv et al., 2021; Maihemaiti et al., 2024) *Bacteroides* species are capable of fermenting polysaccharides and contributing to host energy metabolism (Yuanjia et al., 2021), while *Prevotella*, a common probiotic, has been shown to stimulate glucagon-like peptide-1 release and modulate the gut–pancreas axis (Le Bourgot et al., 2019; Xinya et al., 2023). Therefore, the reduction in these taxa suggests a characteristic dysbiosis in diabetic populations.

The brain-gut axis describes bidirectional communication between the brain and gut *via* neural, endocrine, immune, and metabolic pathways (Zhuang et al., 2024). Increasing evidence suggests that gut microbiota and their metabolites participate in sleep regulation and neurological function (Schneider et al., 2024; Thaiss et al., 2016). In the present study, *TM7x, Achromobacter*, and *Pseudomonas* were higher, whereas *Eubacterium_eligens_group, Lachnospiraceae_UCG-001*, and *Negativibacillus* were lower in diabetic patients with sleep disorders. Previous studies have linked *Achromobacter* and *Pseudomonas* to immune dysregulation and opportunistic infections (Isler et al., 2020; Wen et al., 2022). These three taxa showed higher relative abundances in the sleep-disorder group, but their origin and biological significance require further validation. In contrast, *Eubacterium_eligens_group* and *Lachnospiraceae_UCG-001* are generally regarded as beneficial microorganisms involved in bile acid metabolism, intestinal barrier maintenance, and host metabolic homeostasis (Chen et al., 2017; Sheng et al., 2024). Studies have also linked *Lachnospiraceae_UCG-001* abundance to sleep quality, showing a significant recovery following sleep normalization (Jieming, 2021). Taken together, these findings suggest that sleep disorders in T2DM patients are associated with alterations in gut microbial composition, although the identified genus-level differences should be interpreted as exploratory.

Alteration in metabolites may reflect the complex interaction between sleep disorders and metabolic dysregulation in T2DM (Benz and Forget, 1974). In the present study, a total of 17 nominally differential metabolites were detected between diabetic patients with and without sleep disorders, mainly involving porphyrin- and caffeine-related metabolic features. Among these metabolites, stercobilin, and theobromine were of particular interest as representative metabolites of the two features, respectively.

Stercobilin, a downstream product of heme metabolism, is closely related to porphyrin metabolism and may reflect changes in host metabolic activity and gut microbial metabolism (Hall et al., 2024). Gut microbes participate in the conversion of heme into biliverdin, bilirubin, urobilinogen, and finally stercobilin through a sequence of redox reactions (Sies, 2021). Numerous studies attest to the bile pigment family's endogenous antioxidant properties, and contribute to metabolic homeostasis (Jansen and Daiber, 2012; Ren et al., 2025; Vasavda et al., 2019). In this study, a lower serum stercobilin level was observed in diabetic patients with sleep disorders. Correlation analysis further showed that stercobilin was positively associated with *Odoribacter, Lachnospiraceae_ND3007, and Lachnospiraceae_UCG-001*, suggesting a potential association between gut microbial alterations and metabolite disturbances in T2DM patients.

Caffeine metabolism was detected as another candidate pathway. Accumulating evidence has suggested that caffeine influenced sleep by reducing sleep quality and increasing wakefulness in mice (Ali et al., 2015; Barcelos et al., 2020; Clark and Landolt, 2017; Okuro et al., 2010). Serum theobromine, a major metabolite of caffeine, with milder stimulatory effect on the central nervous system, may serve as a potential candidate feature of caffeine metabolic efficiency (Purkiewicz et al., 2022). In our study, patients with sleep disorders showed decreased serum theobromine level, suggesting a potential alteration in caffeine-related metabolic features. However, circulating theobromine is also influenced by recent intake of coffee, tea, and other caffeine-containing products, and such intake differences alone could produce between-group variation (Clark and Landolt, 2017; Purkiewicz et al., 2022). As we did not document caffeine source, dose, or timing, we cannot distinguish whether the observed reduction reflects intake differences or metabolic alterations. Thus, the caffeine-pathway association remains exploratory, warranting prospective studies with controlled caffeine documentation and comprehensive metabolite profiling.

Several limitations should be acknowledged. First, dietary habits, particularly the amount, source, and timing of caffeine intake, and the use of sleep-related medications were not strictly controlled, and the exact timing of morning sample collection was not standardized. These factors may have influenced the observed microbial and metabolic profiles. Second, the lack of objective sleep monitoring and circadian rhythm assessment, combined with group assignment based solely on subjective scoring, precluded the differentiation of sleep disorder subtypes. Third, the sample size of 40 participants per group provides 80% power to detect a medium-to-large effect size (Cohen's *d* ≈ 0.63) for the primary outcome of gut microbial alpha diversity, but is insufficient for subtler differences and may not provide adequate power for high-dimensional 16S and metabolomic analyses. Fourth, after Benjamini–Hochberg correction for multiple testing, most omics-level differences did not reach statistical significance, supporting the exploratory nature of these findings. Fifth, the single-center design may limit generalizability. Future multicenter studies with larger samples and more comprehensive analytical frameworks are warranted to validate these findings.

In summary, T2DM patients with sleep disorders showed modest differences in gut microbial community structure and exploratory changes in serum metabolite profiles. Exploratory gut microbial changes were characterized by higher relative abundances of potentially inflammation-associated taxa and lower relative abundances of beneficial microorganisms, accompanied by nominal reductions in serum stercobilin and theobromine levels and changes in porphyrin- and caffeine-related metabolites. These findings provide new insights into the gut microbiota-metabolite features associated with sleep disorders among individuals suffering from T2DM and provide a basis for future mechanistic studies.

## Data Availability

The raw 16S rRNA gene sequencing data are available at the National Genomics Data Center (https://ngdc.cncb.ac.cn) under accession PRJCA022517.
