# Supplementary material for: An exploratory study of gut microbiota and serum metabolite profiles in type 2 diabetes patients with sleep disorders
Source: Front Microbiol. 2026 Oct 12;17:1911955. doi: 10.3389/fmicb.2026.1911955 (PMC13696481; doi:10.3389/fmicb.2026.1911955)
Supplement: Supplementary file 1 [file Supplementary_File_1.docx]

**Table S1** Use of glucose-lowering medications in the two groups (n=80).

| Group | Number | Metformin usage rate(%) | Insulin usage rate(%) | Insulin secretagogues usage rate(%) | SGLT-2 inhibitors usage rate(%) |
| --- | --- | --- | --- | --- | --- |
| I | 40 | 20（50.0） | 15（37.5） | 14 (35.0) | 6（15.0） |
| N | 40 | 21（52.5） | 13（32.5） | 8 (20.0) | 8 (20.0) |
| χ2 |  | 0.050 | 0.220 | 2.257 | 0.346 |
| p |  | 0.823 | 0.639 | 0.133 | 0.556 |

Group I: sleep disorder group; Group N: non-sleep disorder group.

**Table S2** Relative abundance and prevalence of *TM7x*, *Achromobacter*, and *Pseudomonas* in Groups I and N (n=80).

| Taxon | Group | Mean relative abundance | SD | Prevalence, n (%) | P value |
| --- | --- | --- | --- | --- | --- |
| *TM7x* | I | 0.0000557 | 0.0001218 | 16 (40.0) | 0.0419 |
|  | N | 0.00001198 | 0.0000527 | 8 (20.0) |  |
| *Achromobacter* | I | 0.0001327 | 0.0001946 | 29 (72.5) | 0.0161 |
|  | N | 0.0000525 | 0.0000576 | 26 (65.0) |  |
| *Pseudomonas* | I | 0.05524 | 0.11846 | 40 (100.0) | 0.0419 |
|  | N | 0.01508 | 0.02495 | 40 (100.0) |  |

Group I: sleep disorder group; Group N: non-sleep disorder group. P values refer to between-group comparisons of relative abundance.

**Table S3** Differential metabolite information between Group N and Group ZC (n=70).

| Number | Name | Formula | FC | Pvalue | ROC | VIP | Trend |
| --- | --- | --- | --- | --- | --- | --- | --- |
| 1 | D-(+)-Mannose | C6 H12 O6 | 2.45 | ＜0.01 | 1.00 | 3.66 | ↑ |
| 2 | ethyl 4-amino-2-(methylsulfanyl)-1,3-thiazole-5-carboxylate | C7 H10 N2 O2 S2 | 2.49 | ＜0.01 | 0.98 | 3.44 | ↑ |
| 3 | (2R,3S,4S,5R,6R)-2-(hydroxymethyl)-6-(propan-2-yloxy)oxane-3,4,5-triol | C9 H18 O6 | 2.48 | ＜0.01 | 0.97 | 3.27 | ↑ |
| 4 | 6-Deoxy-D-glucose | C6 H12 O5 | 0.16 | ＜0.01 | 0.97 | 3.57 | ↓ |
| 5 | Wampetin | C21 H18 O6 | 0.21 | ＜0.01 | 0.95 | 3.41 | ↓ |
| 6 | Dl-Threitol | C4 H10 O4 | 1.64 | ＜0.01 | 0.95 | 3.13 | ↑ |
| 7 | D-(-)-Fructose | C6 H12 O6 | 2.09 | ＜0.01 | 0.97 | 3.21 | ↑ |
| 8 | DL-Norvaline | C5 H11 N O2 | 4.40 | ＜0.01 | 0.95 | 3.10 | ↑ |
| 9 | trans-2-Butene-1,4-dicarboxylic Acid | C6 H8 O4 | 1.63 | ＜0.01 | 0.96 | 3.10 | ↑ |
| 10 | D-Gluconic acid | C6 H12 O7 | 1.96 | ＜0.01 | 0.93 | 2.86 | ↑ |
| 11 | Cyclocytidine | C9 H11 N3 O4 | 1.61 | ＜0.01 | 0.94 | 2.61 | ↑ |
| 12 | D-(+)-Glucose | C6 H12 O6 | 4.05 | ＜0.01 | 0.96 | 2.92 | ↑ |
| 13 | 3-bromo-2,6-dimethoxybenzoic acid | C9 H9 Br O4 | 2.52 | ＜0.01 | 0.95 | 3.03 | ↑ |
| 14 | D-(+)-Galactose | C6 H12 O6 | 2.36 | ＜0.01 | 0.93 | 2.70 | ↑ |
| 15 | Kojic acid | C6 H6 O4 | 1.67 | ＜0.01 | 0.89 | 2.64 | ↑ |
| 16 | D-(-)-Lyxose | C5 H10 O5 | 1.69 | ＜0.01 | 0.87 | 2.45 | ↑ |
| 17 | Acetylcarnitine | C9 H17 N O4 | 2.02 | ＜0.01 | 0.88 | 2.57 | ↑ |
| 18 | Sebacic acid | C10 H18 O4 | 1.74 | ＜0.01 | 0.87 | 2.46 | ↑ |
| 19 | 3-[(methoxycarbonyl)amino]-2,2,3-trimethylbutanoic acid | C9 H17 N O4 | 2.02 | ＜0.01 | 0.87 | 2.60 | ↑ |
| 20 | D-(+)-Proline | C5 H9 N O2 | 1.51 | ＜0.01 | 0.85 | 2.24 | ↑ |
| 21 | SDMA | C8 H18 N4 O2 | 1.51 | ＜0.01 | 0.83 | 2.06 | ↑ |
| 22 | 1-Methyladenosine | C11 H15 N5 O4 | 1.55 | ＜0.01 | 0.84 | 2.35 | ↑ |
| 23 | Tributyl citrate | C18 H32 O7 | 0.03 | ＜0.01 | 0.88 | 2.91 | ↓ |
| 24 | cyclohexyl{4-[4-nitro-2-(1H-pyrrol-1-yl)phenyl]piperazino}methanone | C21 H26 N4 O3 | 0.02 | ＜0.01 | 0.87 | 2.86 | ↓ |
| 25 | N-Acetylhistamine | C7 H11 N3 O | 1.73 | ＜0.01 | 0.93 | 1.63 | ↑ |
| 26 | D-(+)-Maltose | C12 H22 O11 | 2.05 | ＜0.01 | 0.83 | 1.91 | ↑ |
| 27 | S-Sulfo-L-cysteine | C3 H7 N O5 S2 | 2.59 | ＜0.01 | 0.89 | 1.94 | ↑ |
| 28 | N-lactoyl-phenylalanine | C12 H15 N O4 | 1.74 | ＜0.01 | 0.81 | 1.96 | ↑ |
| 29 | 2-methyl-2,3,4,5-tetrahydro-1,5-benzoxazepin-4-one | C10 H11 N O2 | 2.58 | ＜0.01 | 0.78 | 1.82 | ↑ |
| 30 | 2-Methylbutyl beta-D-glucopyranoside | C11 H22 O6 | 0.33 | ＜0.01 | 0.78 | 2.07 | ↓ |
| 31 | (±)9(10)-EpOME | C18 H32 O3 | 0.44 | ＜0.01 | 0.77 | 2.01 | ↓ |
| 32 | 2-imino-8-methoxy-2H-chromene-3-carbonitrile | C11 H8 N2 O2 | 2.59 | ＜0.01 | 0.76 | 1.88 | ↑ |
| 33 | Inositol | C6 H12 O6 | 3.23 | ＜0.01 | 0.87 | 2.18 | ↑ |
| 34 | MAG (18:3) | C21 H36 O4 | 2.04 | ＜0.01 | 0.76 | 1.78 | ↑ |
| 35 | 23-Norcholic acid | C23 H38 O5 | 2.10 | ＜0.01 | 0.77 | 1.72 | ↑ |
| 36 | 1-(4-benzylpiperazino)-2-(pyridin-2-ylamino)propan-1-one | C19 H24 N4 O | 0.39 | ＜0.01 | 0.72 | 1.81 | ↓ |
| 37 | (5S)-5-hydroxy-1,7-diphenylheptan-3-one | C19 H22 O2 | 0.52 | ＜0.01 | 0.71 | 1.81 | ↓ |
| 38 | (+/-)9(10)-EpOME | C18 H32 O3 | 0.43 | ＜0.01 | 0.72 | 1.78 | ↓ |
| 39 | D-Raffinose | C18 H32 O16 | 1.76 | ＜0.01 | 0.72 | 1.53 | ↑ |
| 40 | 4-Butylresorcinol | C10 H14 O2 | 0.60 | ＜0.01 | 0.75 | 1.56 | ↓ |
| 41 | Deoxycholic Acid | C24 H40 O4 | 1.96 | ＜0.01 | 0.75 | 1.67 | ↑ |
| 42 | Asp-Phe | C13 H16 N2 O5 | 2.10 | ＜0.01 | 0.73 | 1.48 | ↑ |
| 43 | Quercetin | C15 H10 O7 | 1.65 | ＜0.01 | 0.83 | 1.72 | ↑ |
| 44 | Trehalose | C12 H22 O11 | 0.29 | ＜0.01 | 0.78 | 1.91 | ↓ |
| 45 | cis,cis-Muconic acid | C6 H6 O4 | 1.57 | ＜0.01 | 0.72 | 1.25 | ↑ |
| 46 | 6-(7-methyloctyl)-1H,3H,4H,6H-furo[3,4-c]furan-1-one | C15 H24 O3 | 0.54 | ＜0.01 | 0.73 | 1.64 | ↓ |
| 47 | 4-acetyl-4-(ethoxycarbonyl)heptanedioic acid | C12 H18 O7 | 0.42 | ＜0.01 | 0.72 | 1.70 | ↓ |
| 48 | 1-[2-(2,5-dimethyl-1H-pyrrol-1-yl)-4-nitrophenyl]-1H-imidazole | C15 H14 N4 O2 | 1.60 | ＜0.01 | 0.71 | 1.43 | ↑ |
| 49 | Sedanolide | C12 H18 O2 | 0.62 | ＜0.01 | 0.71 | 1.63 | ↓ |
| 50 | 2-(2-carboxy-2-methylpropyl)-4,6-dimethylbenzoic acid | C14 H18 O4 | 0.55 | ＜0.01 | 0.72 | 1.49 | ↓ |
| 51 | 4-[2-(2-oxo-1-imidazolidinyl)ethyl]-1lambda~6~,4-thiazinane-1,1-dione | C9 H17 N3 O3 S | 0.63 | ＜0.01 | 0.72 | 1.51 | ↓ |
| 52 | Dihydroroseoside | C19 H32 O8 | 1.64 | ＜0.01 | 0.75 | 1.25 | ↑ |
| 53 | N-Acetylornithine | C7 H14 N2 O3 | 5.11 | ＜0.01 | 0.70 | 1.23 | ↑ |
| 54 | Valproic acid | C8 H16 O2 | 0.51 | ＜0.01 | 0.72 | 1.54 | ↓ |
| 55 | 11-dehydro Thromboxane B2 | C20 H32 O6 | 2.30 | ＜0.01 | 0.72 | 1.31 | ↑ |
| 56 | 2-{1-[2-(4-benzhydrylpiperazino)-2-oxoethyl]cyclopentyl}acetic acid | C26 H32 N2 O3 | 1.79 | ＜0.01 | 0.74 | 1.39 | ↑ |
| 57 | Epitestosterone glucuronide | C25 H36 O8 | 1.52 | ＜0.01 | 0.71 | 1.39 | ↑ |
| 58 | 2-Hydroxy-2-methylbutanedioic acid | C5 H8 O5 | 1.53 | ＜0.01 | 0.77 | 1.50 | ↑ |
| 59 | Chenodeoxycholic Acid | C24 H40 O4 | 1.79 | ＜0.01 | 0.71 | 1.40 | ↑ |
| 60 | 5-[(10Z)-14-(3,5-dihydroxyphenyl)tetradec-10-en-1-yl]benzene-1,3-diol | C26 H36 O4 | 1.77 | ＜0.01 | 0.72 | 1.36 | ↑ |
| 61 | 3-Methylcrotonylglycine | C7 H11 N O3 | 2.06 | ＜0.01 | 0.74 | 1.15 | ↑ |
| 62 | 4-Methylvaleric Acid | C6 H12 O2 | 0.65 | ＜0.01 | 0.72 | 1.45 | ↓ |
| 63 | aminoimidazole carboxamide ribonucleotide | C9 H15 N4 O8 P | 1.56 | ＜0.01 | 0.70 | 1.32 | ↑ |
| 64 | Lauric Acid | C12 H24 O2 | 0.62 | ＜0.01 | 0.71 | 1.32 | ↓ |
| 65 | 3-Hydroxydecanoic acid | C10 H20 O3 | 0.61 | ＜0.01 | 0.71 | 1.45 | ↓ |
| 66 | Esculetin | C9 H6 O4 | 5.35 | 0.01 | 0.68 | 1.30 | ↑ |
| 67 | MLK | C17 H34 N4 O4 S | 1.81 | 0.01 | 0.72 | 1.30 | ↑ |
| 68 | (±)-Abscisic acid | C15 H20 O4 | 0.59 | 0.01 | 0.74 | 1.27 | ↓ |
| 69 | 2-{2-[2,5-di(methoxycarbonyl)anilino]-2-oxoethoxy}acetic acid | C14 H15 N O8 | 1.98 | 0.01 | 0.67 | 1.03 | ↑ |
| 70 | Docosahexaenoic acid | C22 H32 O2 | 0.66 | 0.01 | 0.69 | 1.39 | ↓ |
| 71 | 9-[(2-hydroxyethoxy)methyl]-1,9-dihydro-6H-purin-6-one | C8 H10 N4 O3 | 0.48 | 0.01 | 0.78 | 1.48 | ↓ |
| 72 | Salicylic acid | C7 H6 O3 | 1.59 | 0.01 | 0.67 | 1.04 | ↑ |
| 73 | N-{4-[(2R,3R)-3-(Hydroxymethyl)-5-oxo-2-morpholinyl]phenyl}acetamide | C13 H16 N2 O4 | 1.64 | 0.01 | 0.69 | 1.31 | ↑ |
| 74 | All-Trans-13,14-Dihydroretinol | C20 H32 O | 2.34 | 0.01 | 0.67 | 1.26 | ↑ |
| 75 | N-Phenylacetylglutamine | C13 H16 N2 O4 | 1.61 | 0.01 | 0.69 | 1.30 | ↑ |
| 76 | DL-α-Aminocaprylic acid | C8 H17 N O2 | 0.63 | 0.01 | 0.68 | 1.30 | ↓ |
| 77 | Glycocholic acid | C26 H43 N O6 | 1.93 | 0.01 | 0.69 | 1.26 | ↑ |
| 78 | LMH | C17 H29 N5 O4 S | 3.34 | 0.01 | 0.68 | 1.26 | ↑ |
| 79 | ANH | C13 H20 N6 O5 | 0.48 | 0.01 | 0.67 | 1.34 | ↓ |
| 80 | Dihydrothymine | C5 H8 N2 O2 | 1.55 | 0.01 | 0.69 | 1.22 | ↑ |
| 81 | Royal jelly acid | C10 H18 O3 | 0.52 | 0.01 | 0.67 | 1.68 | ↓ |
| 82 | (2E)-3-phenyl-N-(2-phenylethyl)prop-2-enamide | C17 H17 N O | 1.69 | 0.01 | 0.68 | 1.20 | ↑ |
| 83 | Capric acid | C10 H20 O2 | 0.54 | 0.01 | 0.70 | 1.26 | ↓ |
| 84 | 2-(3-chloro-2-methylanilino)benzoic acid | C14 H12 Cl N O2 | 0.09 | 0.01 | 0.63 | 1.56 | ↓ |
| 85 | 5-hydroxy-6,7-dimethoxy-2-phenyl-4H-chromen-4-one | C17 H14 O5 | 0.38 | 0.01 | 0.67 | 1.49 | ↓ |
| 86 | Syringic acid | C9 H10 O5 | 5.39 | 0.01 | 0.63 | 1.18 | ↑ |
| 87 | D-glutamine | C5 H10 N2 O3 | 1.50 | 0.01 | 0.70 | 1.19 | ↑ |
| 88 | 2,3-Dihydroxybenzoic acid | C7 H6 O4 | 3.57 | 0.01 | 0.61 | 1.10 | ↑ |
| 89 | N5-(2-chloro-6-phenoxybenzyl)-1H-1,2,4-triazole-3,5-diamine | C15 H14 Cl N5 O | 0.60 | 0.01 | 0.74 | 1.28 | ↓ |
| 90 | 12(S)-HETE | C20 H32 O3 | 0.61 | 0.01 | 0.67 | 1.29 | ↓ |
| 91 | 2,3,5,6-Tetramethylpyrazine | C8 H12 N2 | 1219.75 | 0.02 | 0.62 | 1.10 | ↑ |
| 92 | Tetrahydrocorticosterone | C21 H34 O4 | 0.61 | 0.02 | 0.71 | 1.25 | ↓ |
| 93 | DL-Stachydrine | C7 H13 N O2 | 0.55 | 0.02 | 0.67 | 1.39 | ↓ |
| 94 | 23-Nordeoxycholic acid | C23 H38 O4 | 0.65 | 0.02 | 0.67 | 1.14 | ↓ |
| 95 | Glycochenodeoxycholic acid | C26 H43 N O5 | 1.66 | 0.02 | 0.67 | 1.20 | ↑ |
| 96 | Ethyl chrysanthemumate | C12 H20 O2 | 4.06 | 0.02 | 0.66 | 1.10 | ↑ |
| 97 | Rosuvastatin | C22 H28 F N3 O6 S | 5.35 | 0.02 | 0.64 | 1.00 | ↑ |
| 98 | Cholic acid | C24 H40 O5 | 2.34 | 0.02 | 0.65 | 1.10 | ↑ |
| 99 | all-cis-4,7,10,13,16-Docosapentaenoic acid | C22 H34 O2 | 0.66 | 0.02 | 0.67 | 1.33 | ↓ |
| 100 | Cyclamic acid | C6 H13 N O3 S | 3.18 | 0.03 | 0.67 | 1.08 | ↑ |
| 101 | 4-(2,3-dihydro-1,4-benzodioxin-6-yl)-1,2-diphenylbut-2-ene-1,4-dione | C24 H18 O4 | 4.14 | 0.03 | 0.65 | 1.16 | ↑ |
| 102 | Rosmarinic acid | C18 H16 O8 | 5.18 | 0.03 | 0.58 | 1.08 | ↑ |
| 103 | N1-(2,6-dimethylphenyl)-4-aminobenzene-1-sulfonamide | C14 H16 N2 O2 S | 0.65 | 0.04 | 0.65 | 1.05 | ↓ |
| 104 | 4-methylphenyl 1-ethyl-3-methyl-1H-pyrazole-5-carbothioate | C14 H16 N2 O S | 1.72 | 0.04 | 0.66 | 1.13 | ↑ |
| 105 | ACar 18:2 | C25 H46 N O4 | 0.10 | 0.04 | 0.62 | 1.22 | ↓ |
| 106 | Palmitoyl ethanolamide | C18 H37 N O2 | 0.41 | 0.047 | 0.66 | 1.25 | ↓ |
